# *Astragalus* Polysaccharide Suppresses 6-Hydroxydopamine-Induced Neurotoxicity in* Caenorhabditis elegans*

**DOI:** 10.1155/2016/4856761

**Published:** 2016-11-03

**Authors:** Haifeng Li, Ruona Shi, Fei Ding, Hongyu Wang, Wenjing Han, Fangli Ma, Minghua Hu, Chung Wah Ma, Zebo Huang

**Affiliations:** ^1^Center for Bioresources & Drug Discovery and School of Biosciences & Biopharmaceutics, Guangdong Pharmaceutical University, Guangzhou 510006, China; ^2^Research & Development Center, Infinitus (China) Company Ltd., Guangzhou 510665, China; ^3^Guangdong Province Key Laboratory for Biotechnology Drug Candidates, Guangdong Pharmaceutical University, Guangzhou 510006, China

## Abstract

*Astragalus membranaceus* is a medicinal plant traditionally used in China for a variety of conditions, including inflammatory and neural diseases.* Astragalus* polysaccharides are shown to reduce the adverse effect of levodopa which is used to treat Parkinson's disease (PD). However, the neuroprotective effect of* Astragalus* polysaccharides* per se* in PD is lacking. Using* Caenorhabditis elegans* models, we investigated the protective effect of astragalan, an acidic polysaccharide isolated from* A. membranaceus*, against the neurotoxicity of 6-hydroxydopamine (6-OHDA), a neurotoxin that can induce parkinsonism. We show that 6-OHDA is able to degenerate dopaminergic neurons and lead to the deficiency of food-sensing behavior and a shorter lifespan in* C. elegans*. Interestingly, these degenerative symptoms can be attenuated by astragalan treatment. Astragalan is also shown to alleviate oxidative stress through reducing reactive oxygen species level and malondialdehyde content and increasing superoxide dismutase and glutathione peroxidase activities and reduce the expression of proapoptotic gene* egl-1* in 6-OHDA-intoxicated nematodes. Further studies reveal that astragalan is capable of elevating the decreased acetylcholinesterase activity induced by 6-OHDA. Together, our results demonstrate that the protective effect of astragalan against 6-OHDA neurotoxicity is likely due to the alleviation of oxidative stress and regulation of apoptosis pathway and cholinergic system and thus provide an important insight into the therapeutic potential of* Astragalus* polysaccharide in neurodegeneration.

## 1. Introduction

Parkinson's disease (PD) is one of the most prevalent neurodegenerative disorders characterized by progressive loss of dopaminergic neurons in substantia nigra of the midbrain. The dopaminergic neurodegeneration results in severe dopamine depletion and thus leads to a variety of motor complications, including muscle rigidity, tremors, bradykinesia, and postural instability [1]. In the advanced stages of PD, the emerged cognitive and mental problems, such as dementia, depression, and anxiety, further reduce life quality and increase cost burden [2]. Evidences from clinical and epidemiological investigations have demonstrated that the onset and progression of PD is closely associated with aging process. For instance, PD affects approximately 7–10 million people worldwide, and its annualized incidence rate dramatically increases from 0.041% in individuals of 40–49 years old to 0.428%–1.903% in people above 60 years old [3, 4]. Given the detrimental consequences of PD, development of promising therapeutics represents an urgent social and medical need.

A number of studies have revealed that the cause of PD correlates with certain genetic and environmental risk factors, such as mutations in specific genes and toxin exposure [5]. For example, 6-hydroxydopamine (6-OHDA), a toxic oxidation metabolite of dopamine, is detected in the brains and urine of patients with PD [6]. This neurotoxin can selectively enter dopaminergic neurons through dopamine or noradrenaline transporter (DAT and NAT, resp.) and then stimulate overproduction of reactive oxygen species (ROS) via enzymatic oxidation and autooxidation [7]. Increased ROS level elicited by 6-OHDA damages cellular proteins and nucleus, leading to dopaminergic neuron death and dysfunction in PD patients [7]. In experimental models, the administration of 6-OHDA is also able to induce a variety of physiological events including neuronal death and behavioral deficit similar to PD.

*Caenorhabditis elegans* is a relatively simple but powerful animal model in neurobiology field [8]. It has a well-characterized nervous system consisting of 302 neurons, and the neuronal signalling processes such as neurotransmitter formation and release are well conserved. Moreover, exogenous neurotoxin-induced alterations in* C. elegans* are also similar with those in more complex systems. For example, 6-OHDA is capable of degenerating dopaminergic neurons via DAT in both human and* C. elegans* [9]. In addition, genetic manipulation with fluorescent reporter allows for visual inspection of neuronal viability and behavioral performance in the nematodes.

*Astragalus membranaceus* is a well-known Chinese medicine with antiaging functions recorded in Shen Nong's Classic of Materia Medica (~110 BC). It is widely used against mental and emotional stress, anorexia, fatigue, and general weakness [10]. A number of studies have revealed that the neuroprotective effect of* A. membranaceus* is associated with its polysaccharide fraction. For instance, we have recently found that astragalan, an acidic polysaccharide isolated from* A. membranaceus*, is able to reduce the neurotoxicity of polyglutamine, the critical pathogenic protein in Huntington's disease [11].* Astragalus* polysaccharide is also shown to protect rat astrocyte cultures against oxidative damage induced by levodopa, which is widely used in PD treatment but shows adverse effects [12], suggesting a neuroprotective potential of the polysaccharide. However, studies on the underpinning mechanisms of neuroprotection of* Astragalus* polysaccharides in PD models are lacking. Therefore, we investigated the protective effect of the* Astragalus* polysaccharide astragalan against 6-OHDA neurotoxicity using* C. elegans* models and attempted to unravel the underlying mechanisms, including its effects on lifespan, ROS and lipid peroxidation levels, antioxidant enzyme activities, apoptosis-related gene expression, and acetylcholinesterase activity.

## 2. Materials and Methods

### 2.1. Preparation of Polysaccharides

The dry roots of* Astragalus membranaceus* (Fisch.) Bunge were purchased from Tongrentang Group (Bozhou, China). The polysaccharide astragalan was prepared essentially as described previously [11]. Briefly, the sliced roots were refluxed in ethanol, and the materials were used for isolation of* Astragalus* polysaccharides. The polysaccharides were then fractionated by anion-exchange chromatography on a DEAE-Sepharose Fast Flow (GE Healthcare, Uppsala, Sweden) column eluted with water followed by 0.5 M NaCl solution, and the yield of the acidic polysaccharide obtained from NaCl eluate was 1.56%. Further fractionation of the acidic polysaccharide by gel filtration on a Sepharose 6 Fast Flow column exhibited a distinct single peak (data not shown), indicating the homogeneity of the polysaccharide. Thus, the acidic polysaccharide was used as astragalan in the following experiments. The molecular weight of astragalan was measured using high-performance gel permeation chromatography [13], and its number-average molecular weight (Mn) and weight-average molecular weight (Mw) were 427,906 Da and 482,372 Da, respectively. The polydispersity, a ratio of Mw to Mn, of astragalan was ~1.1, indicating a relatively narrow molecular weight distribution of astragalan. Glycosyl composition of astragalan was measured by gas chromatography after methanolysis [14], and the molar composition of monosaccharides in astragalan was 19.2% arabinose, 6.9% rhamnose, 16.0% galactose, 28.8% glucose, and 29.1% galacturonic acid.

### 2.2. Nematode and Bacterial Strains

All* Caenorhabditis elegans* and* Escherichia coli* strains were obtained from the* Caenorhabditis* Genetics Centre (University of Minnesota, Minneapolis, MN, USA). The BZ555 (*Pdat-1*:GFP) nematodes were maintained at 20°C on NGM agar plates with* E*.* coli* OP50 as food. Synchronization was performed using the standard alkaline hypochlorite method as described [15].

### 2.3. 6-OHDA Exposure and Astragalan Treatment

6-OHDA (Sigma, St. Louis, MO, USA) was used to induce selective degeneration of dopaminergic neurons in BZ555 nematodes as described previously [16]. Briefly, synchronized L3 larvae were incubated with 50 mM 6-OHDA and 10 mM ascorbic acid in S. medium supplemented with* E*.* coli* NA22 for 1 h at 20°C. After treatment, the nematodes were washed three times with M9 buffer and then incubated with astragalan at the indicated concentrations in S. medium containing* E*.* coli* NA22 for 24 h. Then 75 *μ*g/mL 5-fluoro-2′-deoxyuridine was added to inhibit reproduction. After further incubation for 48 h, the nematodes were used for various assays. In dopaminergic neurodegeneration, food-sensing behavior, and survival assays, epigallocatechin-3-gallate (EGCG) (final concentration of 0.05 mM) was used as a positive control.

### 2.4. Dopaminergic Neurodegeneration Assay

The nematode dopaminergic neurodegeneration was examined as described previously [16]. In brief, after treatment with 6-OHDA and astragalan, the nematodes were washed with M9 buffer, paralyzed by 50 mM sodium azide, and then mounted onto a 2% agar pad on a glass slide. About 30 nematodes were randomly selected in each treatment, and the GFP fluorescence was imaged using an IX51 inverted fluorescence microscope (Olympus, Tokyo, Japan). The fluorescence intensity in head region was estimated using Image Pro Plus 6.0 software (Media Cybernetics, Rockville, MD, USA).

### 2.5. Food-Sensing Behavior Assay

The food-sensing behavior was carried out as described [16]. Briefly, a 9 cm NGM agar plate was spread with* E. coli* OP50 overnight at 37°C in a ring with an inner diameter of 1 cm and an outer diameter of 8 cm. After treatment with 6-OHDA and astragalan, the nematodes were washed with M9 buffer and transferred to the center of the NGM agar plate with or without bacterial lawn in a drop of M9 buffer, allowed to sit for 5 min, and then the number of body bends of each nematode was counted microscopically for 1 min. The slowing rate was calculated as follows: slowing rate = (*N*_control_ − *N*_food_)/*N*_control_, where *N*_food_ and *N*_control_ represent the numbers of body bends in the plates with and without bacteria lawn, respectively. At least 15 nematodes were scored in each treatment.

### 2.6. Survival Assay

The survival of* C. elegans* was performed using liquid culture at 20°C as described previously [11]. In brief, the nematodes were treated with 6-OHDA and astragalan as above and further incubated in S. medium containing* E. coli* NA22 (initial OD_570_ of ~0.6) and 100 *μ*g/mL ampicillin for 24 h. The nematodes were then transferred into 96-well plates (about 10 nematodes per well). The day of nematode transfer was set as Day 0 in this assay. The numbers of live and dead nematodes were counted microscopically every 2 days based on their movements.

### 2.7. Determination of ROS Level

The ROS level in* C. elegans* was determined using 2′,7′-dichlorofluorescin diacetate (DCFH-DA) (Sigma, St. Louis, MO, USA) as previously described [17]. Briefly, approximately 1000 nematodes treated with 6-OHDA and astragalan were collected, washed, and homogenized in 350 *μ*L of PBS (50 mM, pH 7.8) with 0.1% Tween 20 on ice. The supernatant was collected by centrifugation, and its protein content was determined by BCA assay kit (Thermo Fisher, Waltham, MA, USA). Then 50 *μ*L of the supernatant was transferred into a black 96-well plate and incubated with 50 *μ*L of 100 *μ*M DCFH-DA. The DCF fluorescence was determined in a Fluoroskan Ascent FL microplate reader (Thermo, Waltham, MA, USA) every 10 min for 2 h at an excitation of 485 nm and an emission of 535 nm. The ROS level was calculated as the DCF fluorescence per *μ*g proteins.

### 2.8. Determination of Antioxidant Enzyme Activity and Malondialdehyde Content

The antioxidant enzyme activities and malondialdehyde (MDA) content were determined as previously described [17]. Briefly, about 2,000 nematodes treated with 6-OHDA and astragalan were collected, washed with M9 buffer, and homogenized in 150 *μ*L of PBS (50 mM, pH 7.8) with 1% Triton X-100 and 1 mM PMSF on ice. The supernatant was collected by centrifugation and then used to determine superoxide dismutase (SOD) activity, catalase (CAT) activity, glutathione peroxidase (GPx) activity, and MDA content by the assay kits (Beyotime, Shanghai, China). The protein content of the supernatant was determined by BCA assay kit. The SOD and GPx activities were expressed as U/mg proteins, CAT activity was expressed as U/*μ*g proteins, and MDA content was expressed as nM/mg proteins.

### 2.9. Quantitative Real-Time PCR

Total RNA of the nematodes with or without 2.0 mg/mL of astragalan treatment was isolated using TRIzol reagent (Invitrogen, Carlsbad, CA, USA). After reverse transcription, relative quantification of cDNA by real-time PCR was performed using SYBR Green I (BioRad, Hercules, CA, USA) and MyiQ™2 real-time detection system (BioRad, Hercules, CA, USA) as described previously [11]. Data were normalized to BZ555 nematodes without 6-OHDA exposure and astragalan treatment using the geometric mean of* cdc-42*,* pmp-3,* and* Y45F10D.4* as the reference genes. The primers for PCR analysis are listed in Table 1.

### 2.10. Determination of Acetylcholinesterase Activity

The acetylcholinesterase (AChE) activity of* C. elegans* was determined as previously described [18]. Briefly, approximately 3,000 nematodes with or without 2.0 mg/mL of astragalan treatment were washed twice with M9 buffer and then homogenized in PBS (50 mM, pH 7.8) on ice. The lysate supernatant was collected by centrifugation and then used for determination of AChE activity by the assay kit (Jiancheng Institute of Biotechnology, Nanjing, China). The protein content was determined by BCA assay kit. The AChE activity was expressed as U/mg proteins.

### 2.11. Statistical Analysis

The statistical analysis was performed primarily by GraphPad Prism 5.01 for Windows (GraphPad Software, San Diego, CA, USA). Statistical significance was determined by Student's *t*-test or one-way ANOVA followed by Tukey's* post hoc* test. The survival data were analyzed by Kaplan-Meier method and Peto's log-rank test using SPSS 17.0 for Windows (SPSS, Chicago, IL, USA). A probability value of *p* < 0.05 was considered to be statistically significant. All experiments were performed at least three times.

## 3. Results and Discussion

### 3.1. Astragalan Alleviates 6-OHDA-Induced Neurodegeneration in* C. elegans*

**Figure 1:**
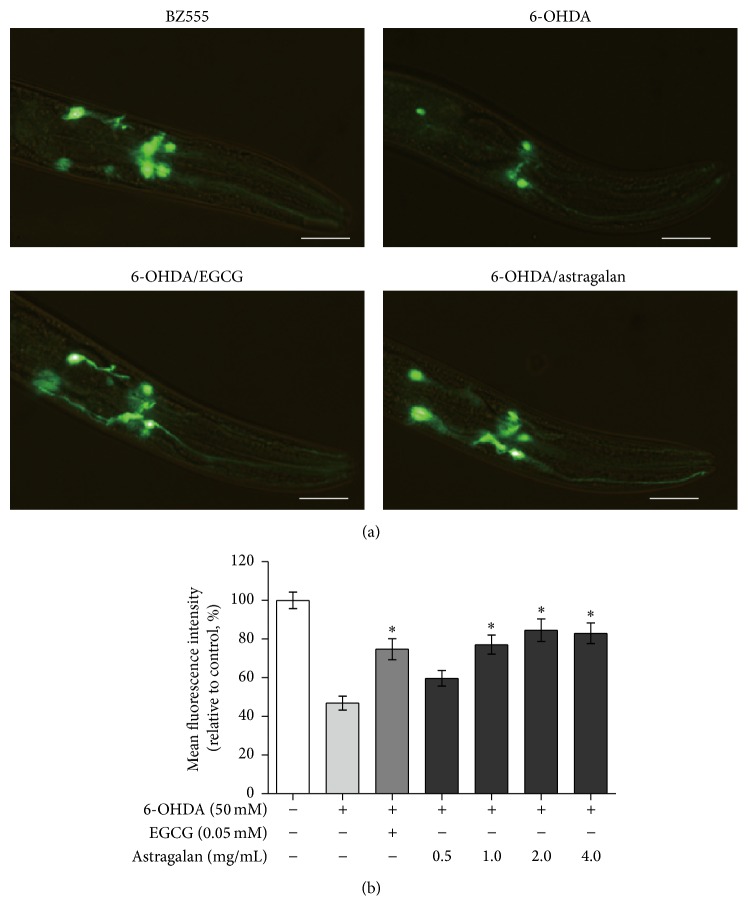
Effect of astragalan on 6-OHDA-induced degeneration of dopaminergic neurons in* C. elegans*. (a) Representative fluorescence images of* Pdat-1::*GFP in dopaminergic neurons were taken from the transgenic* C. elegans* strain BZ555. The nematodes were exposed to 50 mM 6-OHDA for 1 h prior to treatment with astragalan (2.0 mg/mL) or EGCG (0.05 mM). Scale bars, 20 *μ*m. (b) Graphical presentation for fluorescence intensity of* Pdat-1::*GFP in dopaminergic neurons of the nematodes treated with or without astragalan at the indicated concentrations (0.5–4.0 mg/mL). Results are presented as mean ± SEM of three independent experiments. ^*∗*^ *p* < 0.05 versus 6-OHDA-exposed nematodes.

It is well characterized that* C. elegans* contains eight dopaminergic neurons, including two anterior deirid (ADE) neurons and four cephalic (CEP) neurons in the head and two posterior deirid (PDE) neurons in the posterior lateral position [19]. The transgenic* C. elegans* strain BZ555 constitutively expresses GFP in dopaminergic neurons, and the GFP fluorescence intensity indicates the neuronal viability. Previous studies have shown that 6-OHDA exposure reduces the GFP fluorescence markedly in ADE and CEP neurons and weakly in other dopaminergic neurons of* C. elegans* [20]. Therefore, we used this transgenic model to investigate whether the* Astragalus* polysaccharide astragalan was able to inhibit 6-OHDA-mediated neurodegeneration. As shown in Figure 1, in 6-OHDA-exposed BZ555 nematodes, the GFP fluorescence intensity of ADE and CEP neurons was decreased to 46.8% of the unexposed nematodes (*p* < 0.05), demonstrating the capability of 6-OHDA to impair dopaminergic neurons. When the 6-OHDA-intoxicated nematodes were treated with astragalan at the indicated concentrations (1.0, 2.0, or 4.0 mg/mL), the GFP fluorescence intensities were increased to 77.1%, 84.6%, and 82.9%, respectively (*p* < 0.05), which are comparable with the action of the positive control EGCG (74.7% at 0.05 mM), a green tea polyphenol reported to prevent 6-OHDA neurotoxicity [21]. These results demonstrate that astragalan is capable of alleviating 6-OHDA-mediated dopaminergic neurodegeneration in* C. elegans* model.

### 3.2. Astragalan Attenuates 6-OHDA-Induced Food-Sensing Deficit in* C. elegans*

**Figure 2:**
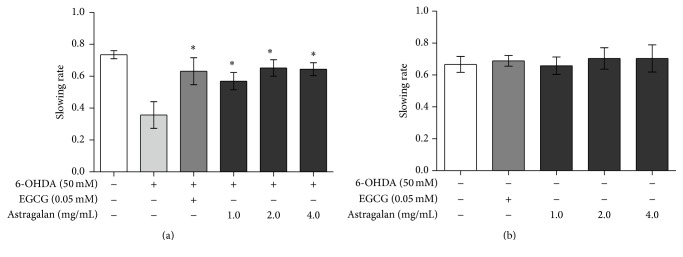
Effect of astragalan on food-sensing behavior in* C. elegans*. (a) The body bends of BZ555 nematodes treated with or without astragalan (1.0–4.0 mg/mL) after 6-OHDA exposure were counted, and the slowing rate was defined as the rate decrease of body bends in the plates with bacteria lawn compared with those without bacteria lawn. (b) The body bends of BZ555 nematodes treated with or without astragalan (1.0–4.0 mg/mL) were counted and used to calculate the slowing rate. Results are presented as mean ± SEM of three independent experiments. ^*∗*^ *p* < 0.05 versus 6-OHDA-exposed nematodes.

Under normal conditions,* C. elegans* moves slowly to sense and consume food. Since the dopamine-mediated neural circuit regulates the basal slowing response of* C. elegans* to food [16], a reduced dopamine level caused by the dopaminergic neurodegeneration makes the nematodes defective in this food-sensing behavior. Since astragalan is able to suppress 6-OHDA-induced dopaminergic neurodegeneration as shown above, we tested whether it could rescue the deficit of food-sensing performance under 6-OHDA exposure. As shown in Figure 2(a), the slowing rate of BZ555 nematodes was 0.73, while 6-OHDA exposure significantly decreased the slowing rate to 0.35 (*p* < 0.05), indicating a deficit of food-sensing behavior due to dopaminergic neuronal dysfunction. When treated with astragalan at the indicated concentrations (1.0, 2.0, or 4.0 mg/mL), the slowing rate of 6-OHDA-intoxicated nematodes was significantly increased to 0.57, 0.66, and 0.64, respectively (*p* < 0.05). As a control, treatment with EGCG also increased the slowing rate to 0.63 under 6-OHDA exposure. However, treatment with astragalan (1.0–4.0 mg/mL) alone had almost no effect on the slowing rate of BZ555 nematodes (Figure 2(b)), indicating that astragalan* per se* did not affect the viability of dopaminergic neurons under normal condition. Together, these data demonstrate that astragalan is able to rescue dopaminergic neuronal dysfunction mediated by 6-OHDA.

It is known that* Astragalus* polysaccharide has antitumor effect against human tumor cells [22]. Interestingly, however, studies have also shown that* Astragalus* polysaccharide has protective effects on neuronal cells [11, 12]. This paradox is likely due to the distinct but also overlapping mechanisms regulated by the polysaccharide under different disease and stress contexts. The anticancer effect of* Astragalus* polysaccharide is most likely due to its immunomodulatory effect rather than direct cytocidal action [23, 24]. For instance,* Astragalus* polysaccharide has shown therapeutic effect by promoting the secretion of proinflammatory cytokines including interleukin and tumor necrosis factor in S180 sarcoma-bearing mice [23]. On the other hand, the polysaccharide can also protect microglial cells against lipopolysaccharide-stimulated inflammation through suppression of nuclear factor-*κ*B and protein kinase B signalling pathways [25]. Interestingly, recent studies have shown that immunotherapy not only is a successful strategy that mediates tumor regression in patients with metastatic cancer but also holds the promise to treat neurodegenerative diseases [26]. Therefore, the specific roles of* Astragalus* polysaccharide in neurons and cancer cells may depend on different cellular and physiological environment.

### 3.3. Astragalan Increases the Lifespan of 6-OHDA-Intoxicated* C. elegans*

**Figure 3:**
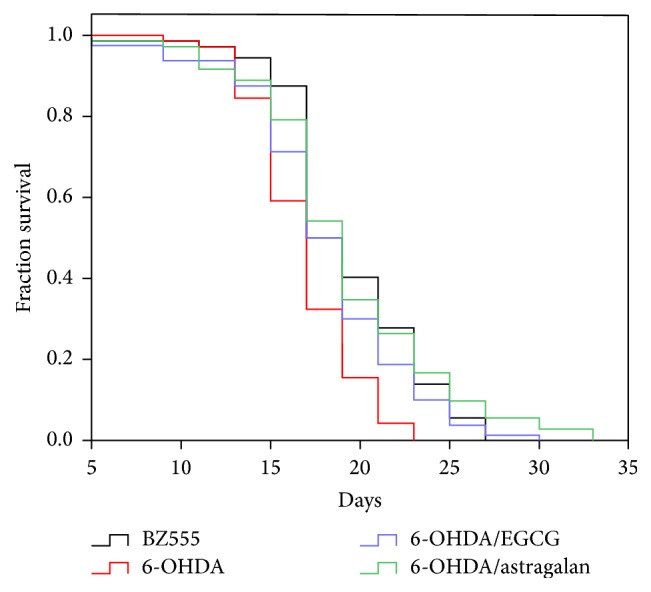
Effect of astragalan on the lifespan of 6-OHDA-intoxicated* C. elegans*. The BZ555 nematodes were treated with or without 2.0 mg/mL of astragalan after exposure to 6-OHDA, and the survival rates were scored every 2 days from the beginning of adulthood until all dead. A representative Kaplan-Meier survival curve from at least three independent experiments is presented.

It is known that aging contributes to the development of late-onset neurodegenerative diseases, and age-dependent neurodegenerative changes are usually associated with a shortened life expectancy [27]. Therefore, we investigated the effect of astragalan on the lifespan of BZ555 nematodes under 6-OHDA intoxication. As shown in Figure 3, 6-OHDA-exposed nematodes exhibited a shorter mean lifespan (16.8 ± 0.35 d) as compared to that of the unexposed nematodes (19.3 ± 0.48 d) (*p* < 0.05), suggesting the toxic effect of 6-OHDA to accelerate senescence. When the 6-OHDA-exposed nematodes were treated with astragalan at 2.0 mg/mL, the mean lifespan was significantly increased to 19.4 ± 0.64 d (*p* < 0.05), which is similar to the action of EGCG (18.1 ± 0.51 d), indicating the capacity of astragalan to delay 6-OHDA-induced senescence. We have previously found that astragalan is able to extend the lifespan of wild-type and transgenic polyglutamine* C. elegans*, and its antiaging activity is associated with the modulation of DAF-16, a major downstream component of the insulin/insulin-like growth factor 1 (IGF-1) signalling pathway related to both lifespan regulation and stress resistance [11]. Recent studies have revealed that manipulation of insulin-related signalling pathways can resist 6-OHDA-mediated neurotoxicity. For example, ginsenoside Rg1 alleviates dopaminergic neuronal injury in 6-OHDA-lesioned rats through IGF-1 receptor, while IGF-1 receptor antagonist JB-1 reduces the neuroprotective effects of ginsenoside Rg1 [28]. Together, these results suggest that regulation of DAF-16 may contribute to the protective effect of astragalan against 6-OHDA neurotoxicity.

### 3.4. Astragalan Reduces ROS Level and MDA Content and Enhances Antioxidant Enzyme Activities in 6-OHDA-Intoxicated* C. elegans*

Oxidative stress is known to play an important role in the pathological process of age-related neurodegenerative diseases. For example, the increase of ROS level and oxidized lipids and proteins was observed in the brains of both sporadic and familial PD patients [29]. The oxidation of 6-OHDA generates cytotoxic hydrogen peroxide and other reactive species, leading to dopaminergic neuronal apoptosis [7]. Therefore, antioxidant intake represents a promising strategy against age-related neurodegenerative disorders [30]. Here we tested whether astragalan was able to reduce ROS level in* C. elegans* under 6-OHDA exposure using the DCF method. As shown in Table 2, the ROS level of 6-OHDA-exposed nematodes was significantly increased as compared to that of unexposed nematodes (*p* < 0.05). When the nematodes were cotreated with 6-OHDA and 2.0 mg/mL of astragalan, the ROS level was reduced compared with that of the nematodes exposed to 6-OHDA alone (*p* < 0.05). When BZ555 nematodes were treated with 2.0 mg/mL of astragalan, the ROS level was also reduced (*p* < 0.05). We then further examined the effect of astragalan on the content of MDA, a toxic lipid peroxidation product. As shown in Table 2, 6-OHDA exposure increased the MDA content in BZ555 nematodes, while astragalan was able to reduce the increased MDA content in 6-OHDA-intoxicated nematodes (*p* < 0.05), indicating its ability to inhibit lipid peroxidation. Together, these data demonstrate the antioxidant capacity of astragalan against 6-OHDA intoxication. Since PD is closely associated with oxidative stress, our results suggest an involvement of antioxidant activity in the neuroprotective effect of astragalan. This is supported by previous studies; for instance, a combination of antioxidant creatine and coenzyme Q10 can delay cognitive function decline and reduce plasma phospholipid level in PD patients [31].

The endogenous antioxidant system, including antioxidant enzymes and nonenzymatic antioxidants, is known to scavenge excessive ROS and maintain cellular redox balance. For example, SOD converts superoxide radicals to hydrogen peroxide and oxygen, while CAT and GPx detoxify hydrogen peroxide to water [32]. Inherited and acquired deficiencies of antioxidant system such as a defect in antioxidant enzyme activity lead to an imbalance of cellular redox environment and thus promote a variety of disorders including PD. For example, inhibition of Cu/Zn-SOD expression and activity aggravates 6-OHDA-mediated neuronal apoptosis [33]. Therefore, enhancing antioxidant system function is helpful to reduce oxidative damage in neurodegenerative diseases. In this study, we examined the effect of astragalan on antioxidant enzyme activities and found that 6-OHDA exposure caused a significant decrease of both SOD and GPx activities in BZ555 nematodes (*p* < 0.05). However, 2.0 mg/mL of astragalan was able to increase SOD and GPx activities in the nematodes with 6-OHDA exposure (*p* < 0.05). It also slightly enhanced CAT activity in 6-OHDA-intoxicated nematodes (Table 2). Interestingly,* Astragalus* polysaccharides are previously shown to scavenge various ROS species* in vitro* and increase the level of nonenzymatic antioxidant glutathione in porcine circovirus type 2 infected cells [34, 35]. Together, these findings indicate that* Astragalus* polysaccharide is capable of enhancing antioxidant system function, and this ability may provide protections against 6-OHDA-induced damage.

### 3.5. Astragalan Suppresses the Proapoptotic Gene egl-1 Expression in 6-OHDA-Intoxicated* C. elegans*

**Figure 4:**
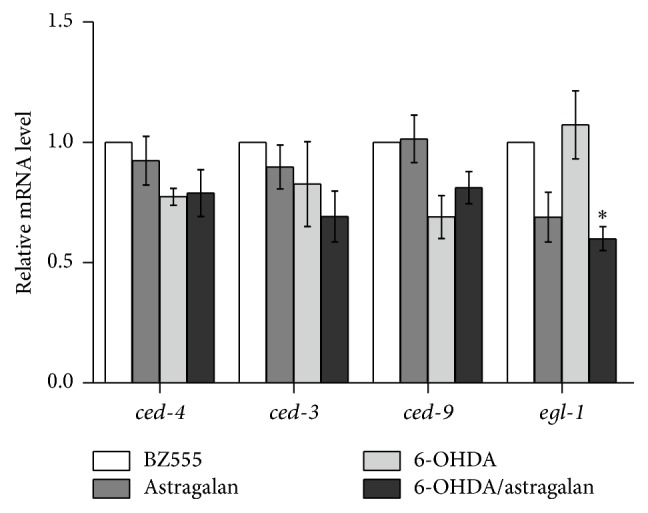
Effect of astragalan on the transcript levels of apoptosis-related genes in* C. elegans*. The mRNA levels of* ced-3*,* ced-4*,* ced-9,* and* egl-1* of BZ555 nematodes with or without 2.0 mg/mL of astragalan treatment under 6-OHDA exposure were performed using quantitative real-time PCR. Data are normalized to the nematodes without 6-OHDA and astragalan treatment and presented as mean ± SEM of three independent experiments. ^*∗*^ *p* < 0.05 versus 6-OHDA-exposed nematodes.

In* C. elegans*, several proteins, including CED-4, CED-3, CED-9, and EGL-1, play a critical role in programmed cell death. The mammalian Apaf-1 counterpart CED-4 promotes the proteolytic activation of CED-3, which is the mammalian caspase-1 homolog and acts as the executor in apoptosis process. This activation process can be inhibited by CED-9, a homolog of the mammalian Bcl-2 and Bcl-XL antiapoptotic proteins. The BH3-only domain protein EGL-1 is a nematode counterpart of proapoptotic Bcl-2 family members. It interacts with CED-9-CED-4 complex and promotes the release of CED-4, resulting in the activation of CED-3 [36]. Previous reports have shown that 6-OHDA exposure induces apoptosis-like changes, such as condensed chromatin structures and shrunken cell morphologies, in dopaminergic neurons of* C. elegans* [37]. To investigate whether the aforementioned apoptosis-related proteins were involved in the actions of 6-OHDA and astragalan, we examined the mRNA levels of* ced-4*,* ced-3*,* ced-9,* and* egl-1* using quantitative real-time PCR. As shown in Figure 4, unexpectedly, 6-OHDA exposure did not influence the transcript levels of* ced-4*,* ced-3*,* ced-9,* and* egl-1* in BZ555 nematodes, suggesting that these gene expressions may be not involved in 6-OHDA-mediated neuronal apoptosis in* C. elegans*. Interestingly, another neurotoxin 1-methyl-4-phenylpyridnium ion (MPP^+^) that can trigger parkinsonism also destroys* C. elegans* dopaminergic neurons independent of* ced-4* pathway [38]. However, a recent study has shown that 6-OHDA activates the mitochondrial apoptosis pathway including the release of cytochrome c to cytosol and the activation of caspase-3 in rat adrenal phaeochromocytoma PC12 cells [39], indicating that other mechanisms may contribute to 6-OHDA-induced neuronal apoptosis. On the other hand, in 6-OHDA-intoxicated nematodes, treatment with 2.0 mg/mL of astragalan exhibited negligible effects on the mRNA levels of* ced-4*,* ced-3,* and* ced-9* but significantly reduced the mRNA level of* egl-1* as compared to those in the nematodes exposed to 6-OHDA alone, indicating a potential of astragalan in regulating apoptotic pathway.

A large body of evidence has shown that modulation of apoptotic pathway holds promise to delay aging and treat aging-related neurodegenerative disorders. For instance, a decrease of* ced-3* expression by overexpression of the mammalian KIF11 homolog* bmk-1* extends the lifespan of wild-type* C. elegans* [40]. The BH3-only domain protein Bim plays a pivotal role in mouse cerebrovascular degeneration induced by *β*-amyloid peptide, the main component of amyloid plaques in patients with Alzheimer's disease, while a decrease of Bim expression attenuates the death of cerebral endothelial cells [41]. Therefore, our data suggest that the regulation of proapoptotic gene* egl-1* expression may contribute to the protective effects of astragalan in* C. elegans*. In addition, a number of signalling pathways play important roles in free radical-mediated cell death [42, 43]. Among these pathways that respond to stresses, the MAPK family, including p38, ERK, and JNK, are important regulators in cell survival and death. Recent studies have shown that MAPKs are involved in the antioxidant and antiapoptotic effects of* Astragalus* polysaccharides. For example,* Astragalus* polysaccharides ameliorate doxorubicin-induced oxidative stress and apoptosis by inhibiting p38 MAPK pathway in cultured primary neonatal rat ventricular myocytes [44]. Thus, the MAPK pathway is likely associated with the protective function of astragalan in 6-OHDA-intoxicated* C. elegans*. Nevertheless, other stress-response signalling pathways may also be involved. For example, a sulfated polysaccharide isolated from sea cucumber* Stichopus japonicus* prevents 6-OHDA cytotoxicity through activation of PI3K/Akt signalling pathway in human neuroblastoma SH-SY5Y cells [45]. However, the exact signalling mechanisms underlying the neuroprotective effect of astragalan need further investigation.

### 3.6. Astragalan Increases Acetylcholinesterase Activity in 6-OHDA-Intoxicated* C. elegans*

**Figure 5:**
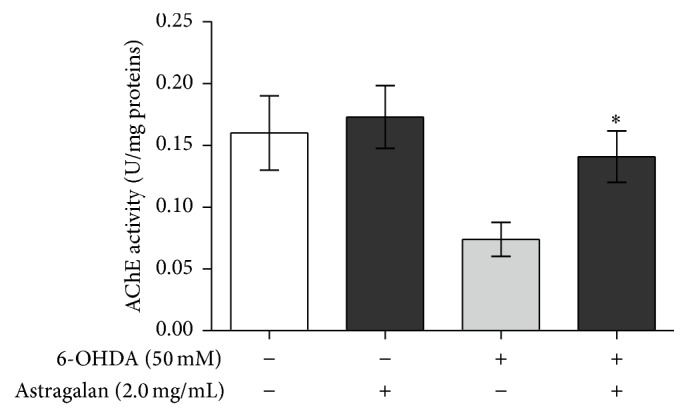
Effect of astragalan on the acetylcholinesterase activity in* C. elegans*. The 6-OHDA-exposed or unexposed BZ555 nematodes were treated with 2.0 mg/mL of astragalan and then used to determine the acetylcholinesterase activities. Results are presented as mean ± SEM of three independent experiments. ^*∗*^ *p* < 0.05 versus 6-OHDA-exposed nematodes.

Although PD is characterized by motor impairment and psychiatric disturbance mainly due to nigrostriatal dopaminergic denervation, increasing evidences reveal that a varying degree of degeneration in the cholinergic, serotonergic, and noradrenergic systems also contributes to motor and nonmotor abnormalities [46, 47]. For example, the nigrostriatal dopamine depletion triggers excessive release of excitatory neurotransmitter acetylcholine, leading to dysfunction of the corticobasal ganglia-thalamocortical loop circuits in PD patients [46]. In* C. elegans*, acetylcholine plays an important role in the control of various behaviors, including locomotion, feeding, mating, and egg laying, whereas acetylcholinesterase (AChE) is able to hydrolyze acetylcholine at neuronal junctions [48]. The administration of 6-OHDA has been reported to decrease AChE activity in animal models such as* C. elegans* and rats [7, 18, 49]. Thus, targeting AChE, an indirect indicator of cholinergic system function, provides a helpful strategy to alleviate the behavioral deficit in PD progression. In the present study, we tested whether astragalan regulated AChE activity in BZ555 nematodes. As shown in Figure 5, AChE activity was decreased in BZ555 nematodes after being exposed to 6-OHDA (*p* < 0.05). When the nematodes were cotreated with 6-OHDA and 2.0 mg/mL of astragalan, the decreased AChE activity was significantly elevated (*p* < 0.05). Interestingly, a previous study has shown that* Astragalus* polysaccharide was able to attenuate homocysteine-mediated inhibition of vasorelaxation to acetylcholine [50], suggesting its potential to influence cholinergic system. Together, our results suggest that the restoration of cholinergic system function may be associated with the protective effect of astragalan against 6-OHDA-mediated neuronal dysfunction.

## 4. Conclusions

In this study, we reveal that* A. membranaceus* polysaccharide astragalan not only attenuates the degeneration of dopaminergic neurons and the deficiency of food-sensing behavior but also increases the lifespan of 6-OHDA-exposed nematodes. We also demonstrate that astragalan is capable of reducing ROS level and inhibiting lipid peroxidation as well as increasing SOD and GPx activities in 6-OHDA-intoxicated nematodes. Further studies indicate that astragalan is able to reduce the transcript level of proapoptotic gene* egl-1* and increase AChE activity in 6-OHDA-exposed nematodes. Together, these findings suggest that the* Astragalus* polysaccharide astragalan can suppress 6-OHDA neurotoxicity through reducing oxidative stress, regulating apoptosis pathway and restoring cholinergic system function.

## Figures and Tables

**Table 1:** List of primers used for quantitative real-time PCR.

	Forward (5′ → 3′)	Reverse (5′ → 3′)
Apoptosis-related genes		
*egl-1*	CTAGCAGCAATGTGCGATGAC	GGAAGCATGGGCCGAGTAG
*ced-9*	TGCTCAGGACTTGCCATCAC	TTGACTCTCCGATGGACATTCTT
*ced-4*	AAGTCGAGGATTAGTCGGTGTTG	AGAGCCATTGCGAGTGACTTG
*ced-3*	TCAACGCGGCAAATGCT	GCCTGCACAAAAACGATTTTC

Reference genes		
*cdc-42*	CTGCTGGACAGGAAGATTACG	CTCGGACATTCTCGAATGAAG
*pmp-3*	GTTCCCGTGTTCATCACTCAT	ACACCGTCGAGAAGCTGTAGA
*Y45F10D.4*	GTCGCTTCAAATCAGTTCAGC	GTTCTTGTCAAGTGATCCGACA

**Table 2:** Effect of astragalan on ROS level, antioxidant enzyme activities and malondialdehyde content in *C. elegans*.

Treatment	ROS level^a^	MDA content^b^	Antioxidant enzyme activity		
			SOD^c^	CAT^d^	GPx^c^
BZ555	4.55 ± 0.20	12.52 ± 2.63	69.35 ± 2.65	1.53 ± 0.09	17.19 ± 0.24
Astragalan	3.60 ± 0.01^e^	9.64 ± 0.90	72.02 ± 6.56	1.48 ± 0.10	19.15 ± 1.29
6-OHDA	7.97 ± 0.55^e^	16.21 ± 4.43^e^	43.90 ± 3.12^e^	1.34 ± 0.07	12.32 ± 0.33^e^
6-OHDA/Astragalan	4.70 ± 0.10^f^	14.39 ± 2.25^f^	69.33 ± 2.46^f^	1.45 ± 0.10	16.44 ± 0.99^f^

^a^ROS level, DCF fluorescence/*μ*g proteins; ^b^MDA content, nM/mg proteins; ^c^SOD and GPx activities, U/mg proteins; ^d^CAT activity, U/*μ*g proteins; ^e^ *p* < 0.05 versus BZ555 nematodes without 6-OHDA exposure and astragalan treatment; ^f^ *p* < 0.05 versus 6-OHDA-exposed BZ555 nematodes.
